# Characterization of the phenotypic and genotypic tolerance to abiotic stresses of natural populations of *Heterorhabditis bacteriophora*

**DOI:** 10.1038/s41598-020-67097-0

**Published:** 2020-06-29

**Authors:** Noa Levy, Adi Faigenboim, Liora Salame, Carlos Molina, Ralf-Udo Ehlers, Itamar Glazer, Dana Ment

**Affiliations:** 1 Department of Entomology and the Nematology and Chemistry units, Plant Protection Institute; Agricultural Research Organization (ARO), the Volcani Center, Rishon Le Zion, Israel; 2 Institute of Plant Science; Agricultural Research Organization (ARO), the Volcani Center, Rishon Le Zion, Israel; 3 The Robert H. Smith Faculty of Agriculture, Food & Environment the Hebrew University of Jerusalem, Rehovot, Israel; 4 e-nema GmbH, Gesellschaft für Biotechnologie und biologischen Pflanzenschutz Klausdorfer Str. 28–36, 24223 Schwentinental, Germany

**Keywords:** Biodiversity, Agroecology, Agricultural genetics, Gene expression, Genome, Genetics

## Abstract

Entomopathogenic nematodes are effective biocontrol agents against arthropod pests. However, their efficacy is limited due to sensitivity to environmental extremes. The objective of the present study was to establish a foundation of genetic-based selection tools for beneficial traits of heat and desiccation tolerance in entomopathogenic nematodes. Screening of natural populations enabled us to create a diverse genetic and phenotypic pool. Gene expression patterns and genomic variation were studied in natural isolates. *Heterorhabditis* isolates were phenotyped by heat- and desiccation-stress bioassays to determine their survival rates compared to a commercial line. Transcriptomic study was carried out for the commercial line, a high heat-tolerant strain, and for the natural, low heat-tolerant isolate. The results revealed a higher number of upregulated vs. downregulated transcripts in both isolates vs. their respective controls. Functional annotation of the differentially expressed transcripts revealed several known stress-related genes and pathways uniquely expressed. Genome sequencing of isolates with varied degrees of stress tolerance indicated variation among the isolates regardless of their phenotypic characterization. The obtained data lays the groundwork for future studies aimed at identifying genes and molecular markers as genetic selection tools for enhancement of entomopathogenic nematodes ability to withstand environmental stress conditions.

## Introduction

Domestication and improvement of crop plants and animals have been part of agriculture for thousands of years. Genetic manipulation of beneficial arthropods, such as silkworms and honeybees, has been conducted for hundreds of years^1,2^ and genetic improvement programs have also provided innovative methods for controlling insect pests^3,4^. Beneficial arthropods have been selected for climate tolerance^5,6^ host–finding ability, host preference^7,8^, improved sex ratio^9,10^, increased fecundity^10,11^, and resistance to insecticides^12,13^. Unlike the long history and vast research on the use of beneficial insects for biological control, the use of entomopathogenic nematodes (EPNs) and the genetic improvement of EPNs is in its infancy. As the use of EPNs for biological control of insect pests becomes practical and commercial due to improvements in production methods^14,15^, the use of powerful genetic tools to improve their performance has been strongly advocated (see reviews ^16,17^).

The only free–living stage of the nematode is the third stage infective juvenile (IJ), a non-feeding larva that lives in the soil, and seeks out and penetrates its host through natural openings^18,19^. The IJ is exposed to changing environmental conditions and its lack of tolerance to extreme environmental conditions directly influences the shelf life, quality and field performance.

Survival, persistence and shelf-life are critical limiting factors for the commercial use of nematodes as biological control agents^20^. These difficulties stem mainly from EPNs’ sensitivity to heat and desiccation stresses^21–23^.

Therefore, two main traits are considered most important for improving: desiccation and heat tolerance. Different approaches have been taken to improve these traits. The first involves a physical/mechanical approach of protecting the nematodes from harmful environmental factors by development of formulation technologies aimed at stabilizing the nematodes during production, distribution and storage^24^. The second approach is screening of wild populations for resistant strains expressing the beneficial traits. An EPNs survey revealed the presence of natural populations of the genus *Heterorhabditis* in arid and semiarid regions in Israel, characterized by high temperatures and dry soils^25,26^. Isolation of natural populations from arid and warm environments indicates the enhanced abilities of some populations to survive harsh environmental conditions^25^. Based on the screening of natural populations, the leading approach to EPN enhancement seems to be a genetic one, through selective breeding of strains with beneficial traits, genetic manipulation (mutagenesis) and genetic engineering^18,27,28^. Advanced genetic techniques, such as gene transformation and induction of mutations using RNA silencing have been studied, and enhancement of beneficial traits for environmental stress tolerance has been reported^25^. However, the genetic study of EPNs requires more advanced information and tools. In a previous study we analyzed the transcriptome expression in *Steinernema* spp. with varied stress-tolerance capabilities and revealed a number of genes and metabolic pathways that are differentially expressed under stress conditions^29^. Still, further transcriptomic and genomic studies under stress conditions are expected to reveal specific genes involved in the stress response, and enable the establishment of expression markers for the enhancement or degradation of beneficial traits.

The main objective of the current study was to establish an infrastructure for the identification of molecular markers associated with heat and desiccation stress in these organisms. This was done by characterizing natural EPN populations for their tolerance to heat and desiccation stress and comparing them with a well-studied commercial line. Based on the phenotypic characterization, isolates with diverse tolerance to the studied stress conditions were chosen for further examination of gene-expression patterns and genomic variations. We hypothesize that: 1. Natural populations of EPNs differ in their tolerance to heat and desiccation stresses. This difference may be related to the characteristics of the isolation site. 2. Gene-expression pattern and genomic sequence differ significantly between isolates with high tolerance vs. low tolerance to environmental stress. To address these hypothesis we followed the following objectives: 1. Isolation of natural populations of EPNs from different habitats and climatic regions in Israel. 2. Phenotyping of heat and desiccation tolerance in these natural isolates, compared to a characterized commercial line. 3. Characterization and comparison of gene-expression patterns in high-tolerance isolates and low-tolerance isolates, using a commercial line as a reference strain for their tolerance capabilities. 4. Identification of genomic variation between high- and low-tolerance isolates, compared to a commercial line.

## Results

### EPN isolation and habitat characterization

**Figure 1:**
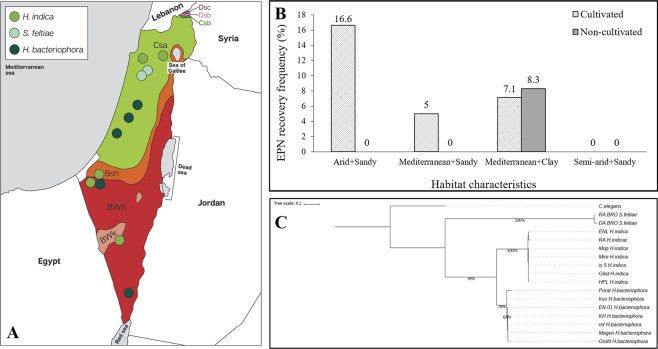
(**A**) Map of Israel divided according to major climatic types based on Köppen climate classification. Dots show the locations at which populations of EPN were isolated in the present study and in previous surveys conducted in Israel (Adobe Illustrator version 24.1.1 https://www.adobe.com/products/illustrator.html). (**B**) Relative frequency of EPN recovery by combinations of three habitat parameters: Soil type (sandy/arid), soil function (cultivated/non-cultivated) and climate (arid/ semi-arid/Mediterranean). χ^2^_(7)_ = 4.92 (P = 0.67). (**C**) Phylogenetic relations of studied isolates from Israel, USA and Germany and a commercial line, EN-01, based on ITS-rDNA sequences as inferred from Maximum Likelihood (ML) analyses. *Caenorhabditis elegans* (MG551717) was used as an out-group. Support values are presented near the nodes in the form: bootstrap in ML.

The occurrence of natural populations of EPN was assessed as recovery frequency (number of positive samples/number total samples) and abundance (number of positive sites/number total sites) expressed as percentage^30^. EPNs were recovered from 8 out of 136 soil samples, 5.8% recovery frequency, from 34 sites, 23.5% abundance. In addition to the overall recovery and abundance of EPN, the relation with soil type, soil function, climate, soil water content and soil temperature was studied. None of the obtained habitat parameters were significant in relation to EPN recovery (χ^2^_(1)_ =0.99 (P = 0.32), χ^2^_(1)_ =0.75 (P = 0.38), χ^2^_(2)_ =1.34 (P = 0.5), χ^2^_(1)_ =0.22 (P = 0.63) and χ^2^_(1)_ =0.01 (P = 0.91) respectively). EPN recovery in relation to soil type, soil function and climate conditions is described as positive samples out of total number of samples in Table 1. Six out of eight positive samples were isolated from cultivated soils of groves and orchards. The two isolates from non-cultivated soils were identified as *Steinernema feltiae*, whereas, all other six isolates were identified as *Heterorhabditis spp*. (Table 1, Fig. 1C). Soil profiling revealed that 50% of the positive samples were from sandy soils and 50% were from clay soils (Table 1). Climate conditions in the different sites, varied from Mediterranean in the northern and coastal regions, and arid or semi-arid in the southern regions (Table 2, Fig. 1A). Relating to three main parameters; climate, soil type and soil function, resulted in eight habitat types. The relative frequency of EPN recovery in relation to those types is described in Fig. 1B. The highest relative recovery frequency (16.6%) was recorded in cultivated habitat with sandy soil and arid climate. Followed by non-cultivated habitat with clay soils and Mediterranean climate (8.3%), which was the only record of EPNs recovered from non- cultivated soils in the present study. Habitat with semi-arid climate and sandy soils did not yielded EPN recovery neither in cultivated soils nor in non-cultivated soils. The water content in the soil samples ranged from 89% in samples collected during January and February, and 10% in samples collected during May. The average water content in soil samples was 21.7% ± 1.8%. EPNs were isolated from soils with minimal water content of 10% and maximal water content of 47%. The average temperature in the soil samples was 20.1 °C ± 0.4 °C, EPN positive samples obtained from soils with minimum temperature of 15 °C and maximum temperature of 23 °C.

**Table 1:** EPN recovery, as percent of positive samples, in relation to three habitat parameters: climate conditions, soil type and soil function.

Habitat parameters		Total samples	Positive samples	% Positive samples	*H. bacteriophora*	*H. indica*	*S. feltiae*
Climate	Arid	16	2	12.5	0	2	0
	Semi-Arid	8	0	0	0	0	0
	Mediterranean	112	6	5.4	2	2	2
Soil type	Sand	83	4	4.8	2	2	0
	Clay	53	4	7.5	0	2	2
Soil function	Cultivated	88	6	6.8	2	4	0
	Non-cultivated	48	2	4.2	0	0	2

**Table 2:** Locations and characteristics of the sampling sites of the present study (sample number 1–34) and previews studies (35–40). bold rows represent sampling sites at which EPNs were isolated.

Sample Number	Region	coordinate	Soil function/vegetation	Soil profile			Climate
				% Sand	% Silt	% Clay	
**1**	**Western Negev**	**34,6680/31,3380**	**Olive grove**	**53.5**	**22.2**	**24.3**	**Arid**
2	Western Negev	34,6689/31,3397	Non-cultivated, *Tamarix* trees	52.5	21.2	26.3	Arid
3	Northern Negev	34,4112/31,2893	Citrus grove	74.5	12.2	13.3	Semi-Arid
4	Northern Negev	34,4209/31,2911	Non-cultivated, *Tamarix* trees	75.5	9.2	15.3	Semi-Arid
5	Western Negev	34,3930/31,2670	Citrus grove	78.5	8.2	13.3	Arid
**6**	**Western Negev**	**34,8750/31,2652**	**Avocado orchard**	**77.5**	**9.2**	**13.3**	**Arid**
7	Menashe Heights	32,35471/35,4541	Avocado orchard	15.9	22.1	62	Mediterranean
**8**	**Menashe Heights**	**32,3548/35,4562**	**Non-cultivated, cypress trees**	**234.9**	**20**	**56.1**	**Mediterranean**
9	Menashe Heights	32,59671/35,0829	Olive grove	19.9	16.5	63.6	Mediterranean
10	Menashe Heights	32,59743/35,0826	Non-cultivated, *Rhamnus*	19.9	20.5	59.6	Mediterranean
**11**	**Menashe Heights**	**32,3652/35,4550**	**Non-cultivated, cypress trees**	**19.9**	**20.5**	**59.6**	**Mediterranean**
**12**	**Menashe Heights**	**32,3651/35,4540**	**Avocado orchard**	**21.9**	**22.5**	**55.6**	**Mediterranean**
13	Jezreel Valley	32,4048/ 35,1136	Olive grove	22.5	12.8	64.7	Mediterranean
14	Jezreel Valley	32,4045/ 35,1152	Non-cultivated, carob trees	22.5	48.7	28.7	Mediterranean
15	Jezreel Valley	32,4120/35,1222	Citrus grove	22.6	8.6	68.8	Mediterranean
16	Jezreel Valley	32,4116/35,1215	Non-cultivated, eucalyptus trees	20.6	12.6	66.8	Mediterranean
**17**	**Lower Galilee**	**32,4222/35,2642**	**Citrus grove**	**22.8**	**20.1**	**57**	**Mediterranean**
18	Lower Galilee	32,4221/35,2645	Olive grove	43.1	19.9	37	Mediterranean
19	Lower Galilee	32,4225/35,2645	Non-cultivated, eucalyptus trees	26.8	16.1	57	Mediterranean
20	Coastal plain	31,5621/34,4633	Annona orchard	85.9	1.6	12.5	Mediterranean
21	Coastal plain	31,5621/34,4633	Non-cultivated, eucalyptus trees	71.7	7	21.2	Mediterranean
**22**	**Coastal plain**	**31,5622/34,4635**	**Citrus grove**	**87.8**	**1.6**	**10.5**	**Mediterranean**
23	Coastal plain	31,5554/34,4629	Olive grove	84.2	3.2	12.6	Mediterranean
24	Coastal plain	31,5552/34,4627	Non-cultivated, eucalyptus trees	71.8	7	21.2	Mediterranean
**25**	**Coastal plain**	**31,5511/34,4637**	**Citrus grove**	**55.7**	**7.3**	**37**	**Mediterranean**
26	Coastal plain	31,5510/34,4637	Non-cultivated	63.3	33.2	3.5	Mediterranean
27	Sharon	32,2152/34,5929	Mango grove	78	6.4	15.7	Mediterranean
28	Sharon	32,2153/34,59303	Non-cultivated	76	6.4	17.7	Mediterranean
29	Sharon	32,14221/34,5560	Citrus grove	86	3.4	10.7	Mediterranean
30	Sharon	32,1414/34,5550	Pomegranate orchards	80	5.4	14.7	Mediterranean
31	Sharon	32,1546/34,5656	Pecan orchard	72	8.4	19.7	Mediterranean
32	Sharon	32,1955/ 35,0207	Mango grove	69	19.7	11.4	Mediterranean
33	Sharon	32,1957/35,0191	Non-cultivated, eucalyptus trees	74	10.4	15.7	Mediterranean
34	Sharon	32,18371/34,5714	Olive grove	88	4.4	7.7	Mediterranean
**35**	**HaArava**	**NA**	**Palm grove**	**sandy**			**Arid**
**36**	**North-West Negev**	**NA**	**Orange orchard**	**sandy**			**Arid**
**37**	**Sharon**	**NA**	**Citrus Grove**	**sandy**			**Mediterranean**
**38**	**Negev**	**NA**	**Avocado orchard**	**sandy**			**Arid**
39	Commercial source	NA	NA	NA	NA	NA	NA
40	Commercial source	NA	NA	NA	NA	NA	NA

In addition to the isolates described above, four isolates from previous surveys conducted in Israel^21,25,31^ and two non-endemic isolates, from USA and Germany were studied. Characterization of collection sites from the present study and from previous studies including isolation sites of all Israeli isolates is presented in Table 2.

### Phylogenetic characterization of studied EPN isolates

Fifteen nematode isolates were characterized at the species level based on the ribosomal ITS region. Electrophoresis revealed 600–800 bp PCR product. BLAST comparison revealed that 5 out of 12 isolates are *H. bacteriophora*, 5 are *H. indica* and 2 are *S. feltiae*. Phylogenetic characterization and relations between the different isolates presented in Fig. 1C and Table 1. A total of 13 *Heterorhabditis* isolates were used in the research, including the commercial line, EN-01. The two isolates belonging to the genus *Steinernema* were not further studied in the present study. The studied nematodes isolates were cultured in the lab during the research period, and freshly emerged IJs were used for all bioassays and molecular studies.

### Host invasion assay as indication for virulence of the nematode isolates

**Figure 2:**
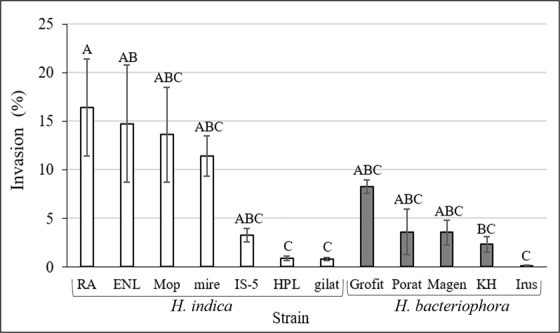
Number of nematodes recovered from the cadavers of last-instar *G. mellonella* larvae following 72 hours of exposure to IJs of the different isolates. Invasion rate in percentage is calculated from the initial number of IJs applied (1000 IJs/5 larvae). Light columns are *H. indica* isolates, dark columns are *H. bacteriophora* isolates. Means of replicates were subjected to One-way-ANOVA followed by post hoc assay of Tukey–Kramer test. Error bars represent standard error for each mean and different letters express significantly different means (P < 0.05).

All isolates caused mortality of the *G. mellonella* larvae within 72 hours. The invasion rate to last-instar *G. mellonella* larvae varied significantly between the different nematodes isolates (F_11, 60_ = 4.39, P < 0.0001) (Fig. 2). The counts of the biological replicates of each experiment were inconsistent, therefore standard errors are high. *H. indica* isolates, RA and ENL displayed the highest invasion rates (16.4% ± 4% and 14.7% ± 6% respectively). The lowest invasion rates were recorded for Irus, Gilat and HPL (0.08% ± 0.05%, 0.8%± 0.15% and 1%± 0.2% respectively).

### Determination of Mean Tolerated Temperature (MT°C50) of commercial line

**Figure 3:**
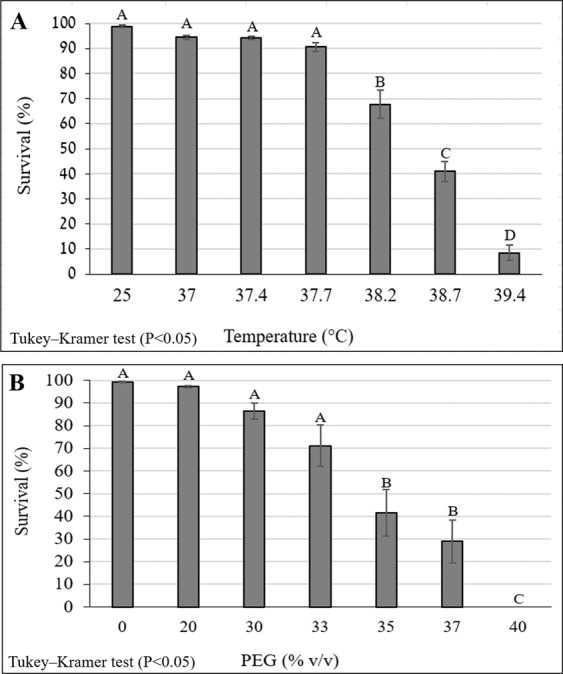
Survival rates (%) of EN-01 IJs under increasing stress conditions. (**A**) Survival rates under increasing gradient temperature. IJs were exposed to adaptation phase of 3 hours at 35 °C, recovery phase of 1 hour at 25 °C and stress phase of 4 hours at gradient temperatures. Survival rates were determined following recovery phase at 25 °C over-night. (**B**) Survival rates under different PEG concentrations (v/v %). IJs were exposed to adaptation phase of 72 hours at 10% PEG solution, followed by stress exposure of 16 hours at gradient PEG dilutions. Error bars represent standard error for each mean and different letters express significantly different means (P < 0.05). Error bars represent standard error for each mean and different letters express significantly different means (P < 0.05).

Since the commercial line EN-01 served as the reference line throughout the study, its mean tolerated temperature was determined and further used for comparison of the studied isolates. Survival rates of IJs of the commercial line, EN-01, decreased with increasing temperature. Survival rates of the control group at 25 °C were 99% ± 0.4. Gradient treatments between 37–39.4 °C had survival rates between 94% ± 0.8% - 9% ± 3%. Tukey’s HSD test revealed significant differences in the survival rates in temperatures higher than 37.7 °C (P = 0.0001). Temperature above 38.2 °C caused more than 50% ± 5% mortality (Fig. 3A). Probit analyses determined the MT_50_°C of EN-01 as 38.8 °C.

### Determination of Mean tolerated Water Activity (MW50) of commercial line

Since the commercial line EN-01 served as the reference line throughout the study, its mean tolerated active water was determined and further used for comparison of the studied isolates. Survival rates of IJs of EN-01 decreased with increasing PEG concentrations at the range of 20–40% v/v equivalent to active water of 0.98–0.96 a_w_. Survival rates of the control group in water were 99% ± 0.3. Tukey’s HSD test revealed significant differences in the survival rates in treatments with PEG percentage higher than 33% v/v (P = 0.03), equivalent to active water lower than 0.97 a_w_. The survival rates in treatments of 0–30% v/v PEG were between 99% ± 0.3%- 70% ± 9%, whereas survival rates in treatment of 35% v/v PEG were 41% ± 10% (Fig. 3B). MW_50_ was determined as 33.88% v/v PEG.

### Comparative heat tolerance in the studied isolates

**Figure 4:**
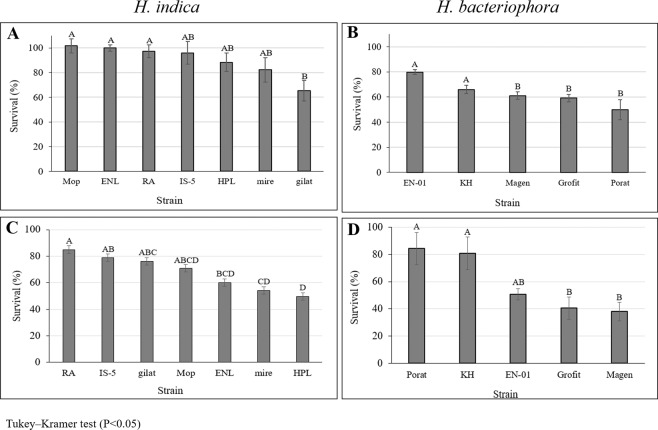
Survival rate (%) under heat stress of 38.8 °C for *H. indica* isolates (**A**) and *H. bacteriophora* isolates (**B**) and desiccation stress of 0.97 aw for *H. indica* isolates (**C**) and *H. bacteriophora* isolates (**D**). Means of replicates were subjected to One-way-ANOVA followed by post hoc assay of Tukey–Kramer test. Error bars represent standard error for each mean and different letters at the top of the columns express significantly different means (P < 0.05). *Reference strain: EN-01, *H. bacteriophora*. Survival rate (%) under.

The mean tolerated temperature determined for the commercial line, EN-01 was used as a standard to compare the survival rates of the isolates in this study. The survival percentages of IJs of the different species and isolates following exposure to heat stress at 38.8 °C is presented in Fig. 4. The results show significant differences (F_11, 117_ = 7.06, P < 0.0001) in the survival rates between the species and among isolates belonging to the same specie. The mean survival percentage was 60% ± 8% and 38% ± 5% among *H. indica* and *H. bacteriophora* isolates respectively. Among *H. indica*, Mop, RA and ENL isolates displayed the highest tolerance, and Gilat isolate displayed the lowest tolerance (Fig. 4A). Among *H. bacteriophora*, the commercial line, EN-01 together with the wild type isolate, KH, displayed the highest tolerance. The other wild-type isolates Grofit, Magen and Porat displayed the lowest tolerance (Fig. 4B).

### Comparative desiccation tolerance in the studied isolates

The mean tolerated active water determined for the commercial line, EN-01, was used as a standard to compare the survival rates of the isolates in this study. The survival percentages of IJs of the different species and isolates following exposure to desiccation stress of 0.97 a_w_ is presented in Fig. 4. The results show significant differences between the two species and among isolates belonging to the same specie (F_11, 132_ = 5.63, P < 0.0001). The mean survival rates of *H. indica* and *H. bacteriophora* were 40% ± 7% and 34% ± 13% respectively. Among *H. indica*, RA displayed the highest tolerance, mire and HPL displayed the lowest tolerance (Fig. 4C). Among *H. bacteriophora*, KH and Porat displayed the highest tolerance, whereas Grofit and Magen showed the lowest tolerance (Fig. 4D). The commercial line, EN-01 did not differ significantly from the highest tolerance isolates (F_4, 55_ = 5.96, P = 0.14, P = 0.08 for differences with KH and Porat respectively).

### Transcriptome mapping

In order to study gene expression patterns under stress condition of heat compared to control conditions, RNA samples were obtained from *H. bacteriophora* isolates with different heat tolerance phenotype. Transcriptome studies using RNA-Seq of the libraries of two isolates of *H. bacteriophora* IJs at early stages of exposure to heat stress and parallel control representing four libraries with three biological replications. The studied isolates were chosen according to the phenotype of survival under heat stress. EN-01 was chosen as a high heat-tolerance isolate and a commercial line and Grofit was chosen as a low heat-tolerance isolate (Fig. 4B). RNA-seq produced approximately 503 million raw reads. The reads were mapped to the reference genome of *H. bacteriophora*, IL3, homogenous inbreed line of the commercial line, EN-01. The average mapping percentage was 87% (Table 3).

**Table 3:** Mapping statistics of *H. bacteriophora* transcriptome.

Sample name	Number per-end reads	%mapped to reference genome
EN01_35 °C	27357421	90
EN01_35 °C	47620031	91.7
EN01_35 °C	34168245	88.3
EN01_ctl	37684891	82.1
EN01_ctl	47458106	66.7
EN01_ctl	28245163	88.3
Grofit_35 °C	22221349	86.8
Grofit_35 °C	57344737	92.6
Grofit_35 °C	46296910	89.2
Grofit_ctl	48594150	87.2
Grofit_ctl	59469487	90.3
Grofit_ctl	47374825	92.5

Principle component analysis (PCA) revealed four clusters representing heat treatment and control for the two studied isolates (Fig. 1S). Each cluster is comprised of three biological replicates of the specific treatment. Results show distinctive clusters of the two heat groups (Fig. 1S: blue and gray dots) compared with the two control groups (Fig. 1S: orange and green dots). However, reads of the heat treatments of both isolates are closely clustered, suggesting similarity in the expression profiles under heat treatment between both isolates.

### Differential expression patterns

**Figure 5:**
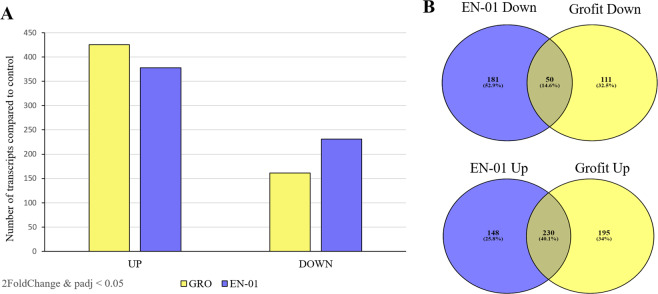
Expression pattern of transcripts during early stages of exposure to heat stress of 35 °C compared to control at 25 °C among *H. bacteriophora* high tolerance strain, EN-01, and low tolerance isolate, Grofit (**A**). Venn diagram illustrating common and specifically differentially expressed, up-regulated transcripts (**B**) and down-regulated transcripts in response to induction of heat stress compared to control among both isolates. Differential expression significance is 2FoldChange&padj<0.05.

During early stages of exposure to heat stress of 35 °C compared to control at 25 °C, a large fraction of transcripts were up-regulated among high tolerance strain (EN-01) and low tolerance isolate (Grofit) (Fig. 5A). 378 transcripts were up-regulated and 231 transcripts were down-regulated in the high tolerance strain, the commercial line EN-01. In the low tolerance isolate, Grofit, 425 transcripts were up-regulated and 161 transcripts were down-regulated. 230 transcripts were commonly up-regulated and 50 transcripts were commonly down-regulated in both isolates (Fig. 5B). As mentioned according to the PCA plot (Fig. 1S), comparison of both isolates under heat treatment, showed similar expression patterns, however, 45 transcripts were identified as differentially expressed (2FoldChange&padj<0.05).

### Putative functional classification using gene ontology (GO enrichment) and KEGG pathway analysis

**Figure 6:**
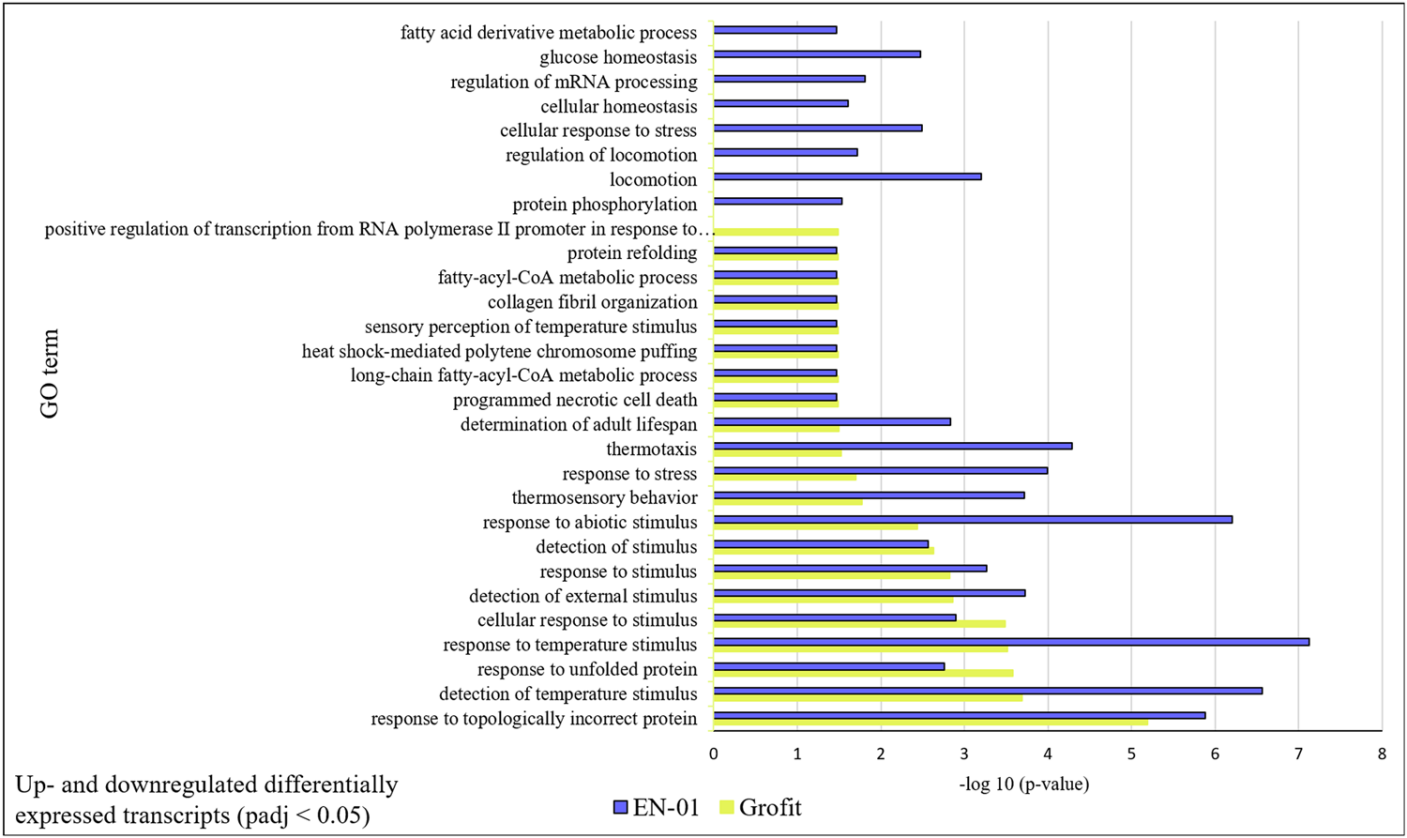
Gene Ontology terms overrepresentation in *H. bacteriophora* high-tolerance strain, EN-01 (white bar) and low-tolerance isolate, Grofit (grey bar) under heat stress. Up- and downregulated genes that changed by twofold and had a significance adjusted p value of less than 0.05 were associated to GO terms. The GO overrepresentation analysis used Revigo. The significance presented as -log10 (P value).

All the differentially expressed transcripts (2FoldChange&padj<0.05) in the comparisons of heat and control treatments within the isolates, and heat treatments between the isolates were analyzed for GO enrichment. Differentially expressed transcripts between heat treatment and parallel control revealed 154 GO terms in the high tolerance strain, EN-01 and 115 GO terms in the low tolerance isolate, Grofit (Table S1). Further analysis revealed 29 enriched GO terms reported as related to stress response in nematodes, including response to topologically incorrect protein, response to heat, membrane organization, programmed cell death, phosphorylation, heat-shock protein activity, protein folding, fatty acid metabolism, response to abiotic stimulus and response to unfolded protein (Fig. 6). The significant difference of the enrichment of those stress-related GO terms between the high tolerance strain, EN-01 and the low tolerance isolate, Grofit is presented in Fig. 6 as -log10 (P value). Overrepresentation of significant (padj < 0.05) enriched GO terms that are related to stress was higher in the high tolerance strain, EN-01. Moreover, enriched GO terms of response to stress, detection of abiotic stimulus, response to temperature stimulus and thermos-sensoring were more significantly differential expressed in the high tolerance strain, EN-01, than the low tolerance isolate, Grofit. Since other genes might be related to stress, yet have not been characterized, further analyses was carried out using the KEGG mapper search tool in order to identify the key metabolic pathways and processes of which the differentially expressed transcripts were mapped to. The functional annotation revealed that 15 of the commonly up-regulated transcripts in both isolates are heat-shock proteins (HSP) related to longevity regulating pathways (Table S2). Two transcripts of the specific down-regulated transcripts of EN-01 were annotated as known heat stress-related proteins, glycerol kinase (GK) and fatty acid desaturase (FAD). Among the up-regulated specific transcripts of EN-01, three were annotated as heat-shock proteins and six were annotated as zinc finger proteins (ZFP), which are also related to stress response (Table 4). Among the specifically down regulated transcripts of the low thermos tolerance isolate, Grofit, two genes were annotated as trehalose coding gene (TRE) as part of the starch and sucrose metabolism (Table 4). Functional annotation of the up regulated transcripts of Grofit did not reveal any stress related pathways and proteins. GO enrichment of the differentially expressed transcripts between Grofit and EN-01 under heat treatment, did not yield significant stress-related GO terms for further evaluation.

**Table 4:** Candidate genes for heat stress tolerance in *H. bacteriophora*, their putative functional annotation according to GO enrichment and KEGG pathway, and SNPs position in.

Transcript ID	GO ID	GO Description	KO ID	KEGG Pathway	Candidate Gene	Expression pattern	#SNPs	Position	High tolerant		Low tolerant	
									IL-3*	KH	Grofit	Magen
mRNA8964	GO:0005991	Trehalose metabolic process	ko:K01194	Metabolic pathway	Trehalose TRE	Down in Grofit	1	4710	G	G/G	G/A	A/A
mRNA11050	GO:0008152	Metabolic process	ko:K01897	Long-chain acyl-CoA synthetase	FAD	Down in EN-01	2	2394	G	G/A	G/G	G/G
								5586	T	T/T	T/A	A/A
Transcript6978	GO:0000166	Diacylglycerol kinase activity	ko:K00901	NA	GK	Down in EN-01	0	—	—	—	—	—
mRNA5799	GO:0015031; GO:0007005	Protein transport; mitochondrion organization	NA	NA	ZFP	Up in EN-01	1	69512	A	A/A	A/G	G/G
mRNA2990	GO:0006915; GO:0043277; GO:0044255	Apoptotic process; cellular lipid metabolic process	ko:K09207	NA	ZFP	Up in EN-01	3	102418	C	C/C	C/G	C/C
								102559	G	A/A	G/A	G/G
								104501	C	A/A	C/A	C/C
mRNA7429	GO:0008233	Peptidase activity	NA	NA	ZFP	Up in EN-01	1	45135	A	A/A	A/T	A/A

*IL-3 - inbreed of the commercial line EN-01.

### Genome sequencing results and SNPs calling

In order to study genomic variation between isolates with varied degrees of stress-tolerance, three *H. bacteriophora* isolates were chosen for genomic sequencing and detection of SNPs. List of the isolates and their determined phenotype under heat and desiccation stress is presented in Table 5. Genomic DNA was sequenced successfully and resulted in 81,269,768, 105,659,854 and 77,291,447 paired end reads for Grofit, KH and Magen respectively. Sequences were mapped to the reference genome of *H. bacteriophora* homogenous inbreed of the commercial line EN-01, IL-3 genome. Mapping percentages are presented in Table 6. The variations among the isolates were identified in comparison with the reference genome, IL-3 regarding homozygous SNPs. There were 6188, 6340 and 8810 homozygous SNPs for Grofit, Magen and KH respectively. Statistics of shared and unique SNPs of each isolate are shown in Table 4, indicating that 2384 SNPs from the Grofit, 2408 SNPs from KH and 3304 SNPs from Magen are located in genes or up/downstream of genes (1000 bp). Additional annotation data of SNPs from each isolate are presented in Table 6. Similarly, 8 SNPs were detected in 5 out of 6 of the differentially expressed stress-related genes up/downstream of genes (1000 bp) (Table 4).

**Table 5:** List of studied *H. bacteriophora* isolates and their phenotypic characterization under stress conditions.

	KH	Grofit	Magen	EN-01 (reference)
Heat stress	High	Low	Low	High
Desiccation stress	High	Low	Low	Low-Medium

**Table 6:** Statistics of *H. bacteriophora* isolates genomic sequencing and summary of annotation data of unique variations containing SNPs.

	Raw reads	% mapped to EN-01 genome	Total SNPs	Total SNPs homozygous	SNPs in genes and up/down stream (1000 bp) of genes	Stop codon	Nonsynonymous
Grofit	81,269,768	84.48	46,408	6188	2384	46	365
KH	105,659,854	25.5	27,057	6340	2408	46	366
Magen	77,291,447	66.78	55134	8810	3304	54	488

### Analysis of variation between isolates compared to the reference genome

**Figure 7:**
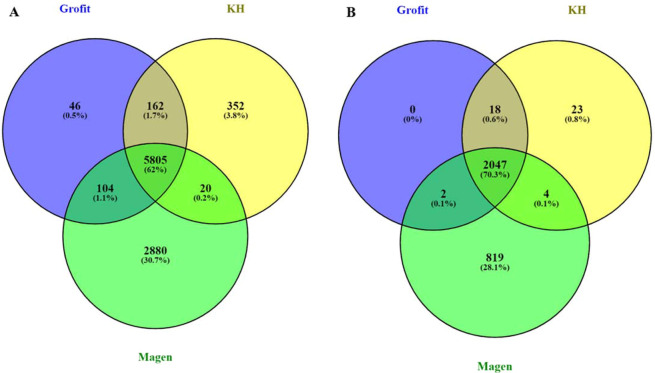
(**A**) Venn diagram of specific and unique homozygous SNPs between *H. bacteriophora* isolates Grofit, KH and Magen compared to the reference genome, IL-3. (**B**) Venn diagram of specific and unique homozygous SNPs in genes sequences and up/downstream (1000 bp) from genes, between *H. bacteriophora* isolates Grofit, KH and Magen compared to the reference genome, IL-3.

In order to compare the genomic variation between the studied isolates, homozygous SNPs positions of each isolate compared to the reference genome, were analyzed for common and unique SNPs. Results revealed that Magen isolate had the highest number of unique SNPs position (2880 SNPs, 30.7% of total positions). Followed by KH with 352 unique SNPs and Grofit with the lowest number of unique SNPs (46 SNPs, 0.5% of total positions) (Fig. 7A). Further analysis of common and unique genes containing SNPs revealed similar trend, as Magen had the highest number of unique SNPs in genes (819 genes), followed by KH with 23 unique SNPs in genes and Grofit with no unique SNPs located in genes (Fig. 7B).

## Discussion

The results stem from both transcriptomic data for differential expression between isolates with high vs. low stress tolerance, and analysis of genomic data for SNPs among specific isolates with different degrees of stress tolerance. The obtained information contributes to our understanding of the differences between isolates with varied degrees of abiotic stress tolerance and to a future establishment of molecular markers to breed strains with improved performance.

### Isolation and characterization of natural EPN populations

To enrich the genetic pool, natural EPN populations were collected from different habitats across Israel. Abiotic factors are known to have strong effects on EPN occurrence, persistence and species variation^32–34^. However, previous studies conducted worldwide have shown varied correlations between effects of habitat parameters and EPN occurrence^35–37^. Initially, the recovery frequency and abundance of EPNs were evaluated according to the number of positive samples out of the total number of samples and collection sites. The results (5.8% recovery frequency and 23.5% abundance) were similar to recovery rates reported in previous surveys conducted in Israel and in other Mediterranean areas^30,31,38–40^. The sampling period (January to May) was considered part of the rainy season in Israel, which extends from October to early May. However, rainfall peaks are common in December through February and rainfall varies considerably by region from north to south. To maximize the potential for EPN isolation, sampling was performed close to rainfall events and under tree canopies. To investigate the effect of habitat characteristics on the occurrence of EPNs, the collection sites were deliberately selected in different climatic regions, and in each region, samples were collected from cultivated and non-cultivated sites. Soil profiling of sand, silt and clay content revealed two main types of soil related to the climatic region: high clay soils were found only in Mediterranean regions, whereas high sand soils were found in arid and Mediterranean regions. Therefore, to study the relationship with EPN occurrence, the parameters of climate and soil type were integrated, producing four habitat types. Additional integration with the soil function parameter resulted in eight habitat types according to three parameters: climate, soil type and soil function. The occurrence of natural EPN populations was evaluated in relation to these habitat types, but none of the parameters were found to be significant. Nevertheless, certain trends corresponding with previous EPN surveys were recorded. Overall, the number of positive samples was higher in soils from cultivated sites compared to non-cultivated sites, in agreement with surveys reporting high recovery rates of EPNs from orchards and groves in the United States, Portugal and Israel^36–38,40^. Moreover, species variation was found among cultivated and non-cultivated sites. Isolates belonging to the species *S. feltiae* were only recovered from non-cultivated sites, whereas *Heterorhabditis* species were recovered only from cultivated soils. These findings might be explained by the foraging strategy of *Heterorhabditis* species, which are known to disperse deeper in the soil compared to ‘ambusher’ species of the genus *Steinernema*; they are therefore more influenced by tillage and agronomic practices^18,41^. Another interesting trend was that *Steinernema* isolates were only recovered from Mediterranean habitats, whereas *Heterorhabditis* isolates were recovered from arid as well as Mediterranean habitats. Nematodes of the genus *Heterorhabditis* are known to exist in habitats with a warm and arid climate, compared to *Steinernema* which are more abundant in humid and high-altitude habitats, usually characterized by lower temperatures^18,37,42^. Additional habitat parameters, such as soil temperature and altitude, showed minor differences between collection sites, and were therefore found irrelevant for the analysis of EPN occurrence.

In the current study we provide a taxonomic classification of naturally occurring Israeli EPNs to the species level, using molecular tools based on the ribosomal ITS region. Six *Heterorhabditis* isolates and two *Steinernema* isolates were identified. Another four isolates from previous studies were molecularly identified. Phylogenetic analyses revealed that the isolates belong to three different species: *H. bacteriophora*, *H. indica* and *S. feltiae*. This is the first confirmed report of *H. indica* in Israel, a species that was first reported in India and is widely distributed across all continents except for Antarctica. It has been reported as a dominant species in citrus groves in Florida, as well as in coastal regions in other Caribbean locations^38^. Campos-Herrera *et al*. (2019) recently published a first report of *H. indica* in a Mediterranean region, in a survey conducted in Portugal. Species variety in the present study was low compared to previous studies of natural populations worldwide^32,34,37,38,43^. This might be due to aggregated spatial distributions of the species, which is a well-known distribution pattern for EPNs^18,33,35^. The patchy distribution might also provide a reasonable explanation for the lack of association of habitat parameters with EPN occurrence^30^. For ecological research purposes, this must be taken into account by randomly collecting a larger number of samples from each site.

### Characterizing heat and desiccation tolerance of natural isolates of Heterorhabditis compared to a commercial line

Application of EPNs for biocontrol is restricted to habitats with favorable microenvironments, such as soil and cryptic habitats that provide protection from extreme environmental conditions^21,44^, unless applied with protective formulations. To increase the use of EPNs as biocontrol agents, the main objective is to overcome their intolerance to heat and desiccation stress. In the present study, the survival properties of natural isolates under extreme heat and desiccation conditions was evaluated in comparison to the commercial line EN-01. As the tolerance properties of this latter strain have been studied, it was chosen as a reference strain for the characterization of the tolerance capabilities of native populations from Israel. For the present study, we determined the MT_50_°C and MW_50_ of EN-01. The MT_50_ was 38.8 °C, and the MW_50_ was 0.97_aw_, indicating high heat tolerance and low to medium desiccation tolerance according to previous studies^20,45^. Phenotyping of the natural *H. bacteriophora* isolates under heat stress revealed three isolates with significantly lower tolerance and one isolate with similar tolerance compared to the commercial line EN-01. Under desiccation stress, none of the natural isolates displayed significant differences in tolerance compared to EN-01.

As the main objective of the research was to establish genetic selection tools based on the phenotypic characterization of natural EPN populations, we needed to obtain a wide range of phenotypic characteristics. We hypothesized that natural isolates will differ in their tolerance to heat and desiccation stresses. This hypothesis was confirmed by the variations in survival rates among species and among isolates within species. In general, *H. indica* isolates displayed higher survival rates than *H. bacteriophora* isolates. Such variations in tolerance among isolates and species have been reported previously; moreover, *H. indica* has been reported to be a high heat- and desiccation-tolerant^21,27,46,47^. It should be noted that the phenotypic characterization of stress tolerance in the present study was determined according to a single parameter—survival rate under stress—determined by stereoscopic observation. Further evaluation of morphological characteristics, virulence and reproduction of the surviving nematodes may provide valuable information on their tolerance capabilities.

### Gene-expression patterns under heat stress in H. bacteriophora isolates

RNASeq was only carried out for *H. bacteriophora* heat-exposed and control samples. This enabled comparing the transcriptomic and genomic data to the existing data on the commercial line EN-01. Isolates with significantly different survival rates under heat stress were chosen for RNASeq analysis in search of variation corresponding to the phenotypic differences. We hypothesized that gene expression would differ significantly between isolates with varied degrees of tolerance to environmental stress. The genes that varied the most were selected as candidate markers for the stress-resistance trait. As already noted, the commercial line EN-01 is characterized by high heat tolerance. This is the first study to provide transcriptomic data for this strain. The Israeli isolate, Grofit, was isolated in the Arava region and was phenotyped as having low heat tolerance in the present study. A comparison of gene expression in these two strains under heat stress of 35 °C vs. control conditions of 25 °C revealed a higher number of upregulated transcripts than downregulated transcripts in both strains. However, a comparison of the numbers of expressed transcripts revealed different expression levels between the strains. The high heat-tolerant strain EN-01 expressed both a lower number of upregulated transcripts and a higher number of downregulated transcripts, compared to the low heat-tolerant isolate Grofit. Previous studies have shown a similar relationship between gene-expression patterns and stress-tolerance capabilities, i.e., downregulation of transcripts in strains with high tolerance and upregulation of transcripts in those with low tolerance^28,29^. Although the overall expression pattern of both strains in the present study was upregulation, the results correspond with those previous findings.

### Specific stress-related genes

The analysis of gene ontology and pathways of the differentially expressed transcripts revealed common expression of certain heat-shock proteins (HSPs) and related pathways of HSP activity, regardless of the strains’ survival rates, supported by reports of HSP synthesis as a common response to stress^28^. Transcripts expressed only in the high heat-tolerant strain EN-01 were annotated as known stress-related genes, encoding glycerol kinase (GK), fatty acid desaturase (FAD) and zinc finger protein (ZFP). These three genes have been previously reported with similar expression patterns under heat stress, suggesting high suitability as candidate expression markers for heat tolerance^14,29,48,49^. Functional annotation of the uniquely downregulated transcripts of the low heat-tolerant isolate Grofit revealed the known stress-related gene encoding trehalose (TRE). High temperature is known to trigger the accumulation of trehalose in EPNs and the accumulated trehalose correlates with enhanced heat and desiccation tolerance^14^. These findings suggest downregulation of the trehalose-encoding gene as a candidate expression marker for the identification of low heat-tolerant strains.

### Genomic variation

Identification of genomic variation between high and low stress-tolerant isolates might be used as a foundation for selection markers based on polymorphism in the genome. Natural EPNs populations have highly diverse phenotypes and genotypes^20,47^. In the present study, the genomes of three natural isolates of *H. bacteriophora* were sequenced to explore the genomic differences between them. We hypothesized that the genomic sequence would differ significantly between isolates with high tolerance vs. low tolerance to environmental stress. However, the results only partially supported this hypothesis. Two of the isolates, Magen and Grofit, were characterized as having low tolerance to heat and desiccation. The third isolate, KH, was characterized as having high tolerance to heat and desiccation, and the reference strain had high heat tolerance and low to medium desiccation tolerance. Magen was found to be the most distant isolate from the reference, whereas Grofit was most similar to the reference line EN-01. As Magen and Grofit have the same low-stress-tolerance phenotype, this result did not support the hypothesis. However, the distance of Magen (low tolerance) from KH (high tolerance), as well its distance from the reference strain, indicated distinct genomic differences between isolates with varied degrees of stress tolerance. Since the *H. bacteriophora* genome is still in its assembly version represented as scaffolds, we could not create a circus map to compare SNPs positions among isolates^50^. But, this comparison enabled the identification of common SNPs in the natural isolates which can be further studied for their association with phenotypic characterizations of EPNs. We identified 8 SNPs in the candidate genes for heat stress tolerance. Part of these SNPs were polymorphic between homozygous isolates. It is important to note that genotype–phenotype associations in natural populations using the single phenotypic parameter of survival is not ideal. The main objective of this work was to establish a basis for further research by screening natural populations and identifying various positions in their genomes that are potentially suitable for use as molecular markers for beneficial traits.

### Further research and use of the obtained knowledge

The present study provides a phenotypic characterization of the survival rates of natural populations of EPNs belonging to two species: *H. bacteriophora* and *H. indica*. The phenotypic variations led to variations in the nematodes’ survival rates. To identify the genotypic variations, the isolates that varied the most in their tolerance to each of the studied stress conditions were chosen for transcriptomic comparison. The results presented here are for two *H. bacteriophora* strains under heat stress. Several differentially expressed genes between the highly heat-tolerant strain EN-01 and the low heat-tolerant isolate Grofit were identified as stress-related genes reported in previous studies. These genes were chosen as candidate marker genes. Previous studies of EPNs under heat and desiccation stress have revealed that IJs use a conserved stress-tolerance response that may be triggered by changes in temperature or desiccation regime. Once this stress response is induced, the nematodes acquire resistance to multiple factors, including heat, desiccation and ultraviolet radiation^51^. Therefore, further study of the transcriptome of *H. bacteriophora* isolates under desiccation stress and *H. indica* isolates under heat and desiccation stress will provide additional important information regarding the expression patterns of stress-related genes under stress conditions and their suitability for use as expression markers for beneficial traits of multiple-stress resistance in EPNs. Eventually, the candidate genes will be further validated for use as expression markers using RT-qPCR under stress conditions. By studying the genomic sequence variations between natural isolates with varied degrees of stress tolerance, specific SNPs were found that may be associated with beneficial traits. Future study of the specific physical and functional positions of those SNPs along the *H. bacteriophora* genome will provide important information on their relevance as markers.

## Conclusion

Natural populations of EPNs were isolated from different habitats across Israel and characterized at the species level, phenotypic level (degree of tolerance to heat and desiccation stress) and genotypic level (variations in gene expression and genome sequence). The phylogenetic analysis revealed three EPN species, *S. feltiae*, *H. bacteriophora* and *H. indica*, the latter reported for the first time in Israel. The phenotypic characterization of the different isolates under stress conditions revealed different degrees of tolerance in terms of survival after a defined period of stress. Pursuant to the main objective of the study, strains with the most different survival properties were chosen for genotypic characterization. This characterization revealed differences in the expression of specific known stress-related genes that need to be further validated. Furthermore, genomic sequence variations between natural isolates with varied degrees of stress tolerance were identified. Among these variations, specific SNPs were found which may be associated with beneficial traits. This study is the first to provide a phylogenetic characterization and genomic information on natural EPN populations in Israel, in comparison to a well-studied commercial line. The obtained knowledge lays the groundwork for future studies aimed at identifying molecular markers as genetic selection tools for enhancement of EPNs’ ability to withstand environmental stress conditions.

## Materials and Methods

### Soil samples collection and characterization

During the rainy season of January to May 2018, 136 soil samples were collected from 34 sites across Israel (Fig. 6). Each site was of 100 m^2^ cultivated or non-cultivated land. At each site, four samples were collected into pots from a distance of 30–40 cm from the tree trunks, under the canopy, at 15–20 cm depth using hand shovel. Each sample contained 500 gram of soil. Three of the samples were subjected for EPN isolation and the fourth sample was subjected to water content analysis and soil profiling. Water content analysis was performed according to the gravimetric method^52^. Soil samples were air-dried for one week in room temperature. The differences between the weight of wet soil and the weight of dry soil (g) were calculated as the mass of water in the sample. The gravimetric water content was calculated as the mass of water per mass of dry soil, presented as percentages. Soil profiling of sand, silt and clay content was done at the Field Service Lab, Neve Ya’ar. Soil temperature was measured at each site using a thermometer probe inserted to the soil at 10 cm depth. Soil function (cultivated or not) and the type of vegetation were recorded. Climate characteristics were obtained from the Israel Meteorological Service (IMS) (http://www.ims.gov.il), according to the annual precipitation.

### Isolation of EPNs from soil

Isolation of nematodes was done selectively for entomopathogenic nematodes using a baiting technique as described before by *Galleria mellonella* (L.) larvae obtained from a lab colony at Volcani center^31,53^. One trap with 5 larvae was inserted to each soil sample and samples were incubated at 25 °C for one week. At the end of incubation, mortality rates of larvae in the traps were recorded. Dead larvae were further incubated in white traps at 25 °C in order to collect emerging IJs from the insect cadavers, as described by^54^. Emerging IJs were collected in water and stored in a Flask tissue culture at 12–14 °C.

### Nematode culture

Recovered IJs from the white traps were re-exposed to last-instar *G. mellonella* larvae in a 50 cm diameter petri dish lined with moist filter paper. New generation of emerging IJs were collected in Flask tissue culture and kept in 12–14 °C for further research. Nematodes rearing during the research was done similarly, by infection of last-instar *G. mellonella* larvae with freshly emerged IJs every two weeks as described by^54^.

### Molecular characterization and identification

Nematodes in water suspension were poured into 15 ml tubes and precipitate for 1 hour until a concentrated residue of nematodes was in the bottom of the tube. Two-hundred μl, equivalent to about 1000 IJs, were transferred to 2 ml tubes and lyophilized overnight. Dried samples were ground with the Geno/Grinder 2,010 (SPEX SamplePrep, New Jerzey, USA). DNA extraction was performed by the DNeasy Blood & Tissue Kit (QIAGEN). PCR amplification of the Internal Transcribed Spacer region (ITS) of the ribosomal DNA (rDNA), containing ITS1, 5.8 s and ITS2 was performed with the following primers (Iqbal, Ehlers, & Waeyenberge, 2016):

Forward Primer: AB28 (5′-ATATGCTTAAGTTCAGCGGGT-3′)

Reverse Primer: TW81 (5′- GTTTCCGTAGGTGAACCTGC-3′).

Amplified products at size of 600–800 bp were sequenced by Sanger method. Sequences were compared with ITS sequences in the GenBank by means of BLAST in NCBI and WormBase Parasite database. The ITS sequences of the various isolates were compared using the MAFFT software (Version 7, http://mafft.cbrc.jp/alignment/server/). Bootstrap values were calculated by 100 repetitions as described by^55^. Phylogenetic tree, describing the phylogenetic relations between the various nematodes isolates was designed using TOL software (https://itol.embl.de/).

### Host invasion rate assay

The infectivity rate of the different EPN isolates was determined by invasion host assay as described by Glazer and Lewis (2000). Five last-instar *G. mellonella* larvae were placed in a 50-cm-diameter petri dish lined with moist filter paper. A thousand IJs of each isolate were added to each petri dish and the dishes were incubated at 25 °C for 72 hours to ensure sufficient time for nematodes invasion and adult hermaphrodite development inside the larvae cadavers. Subsequently, larvae were washed in tap water in order to remove any nematodes from the outer surface. Larvae cadavers were dissected by scalpel under a stereomicroscope and tap water were added to the plates in order to encourage movement of the invading nematodes, for better visualization. The invasion rate of each nematode isolate was determined according to the number of nematode counted in each plate and presented as percentage of invasion out of the initial number of nematodes applied in each plate (1000 IJs per 5 larvae). Three replicates were carried out for each nematode isolate (one petri dish = one replicate) and the experiment was repeated three times.

### Heat tolerance bioassay – Gradient temperature

**Figure 8:**
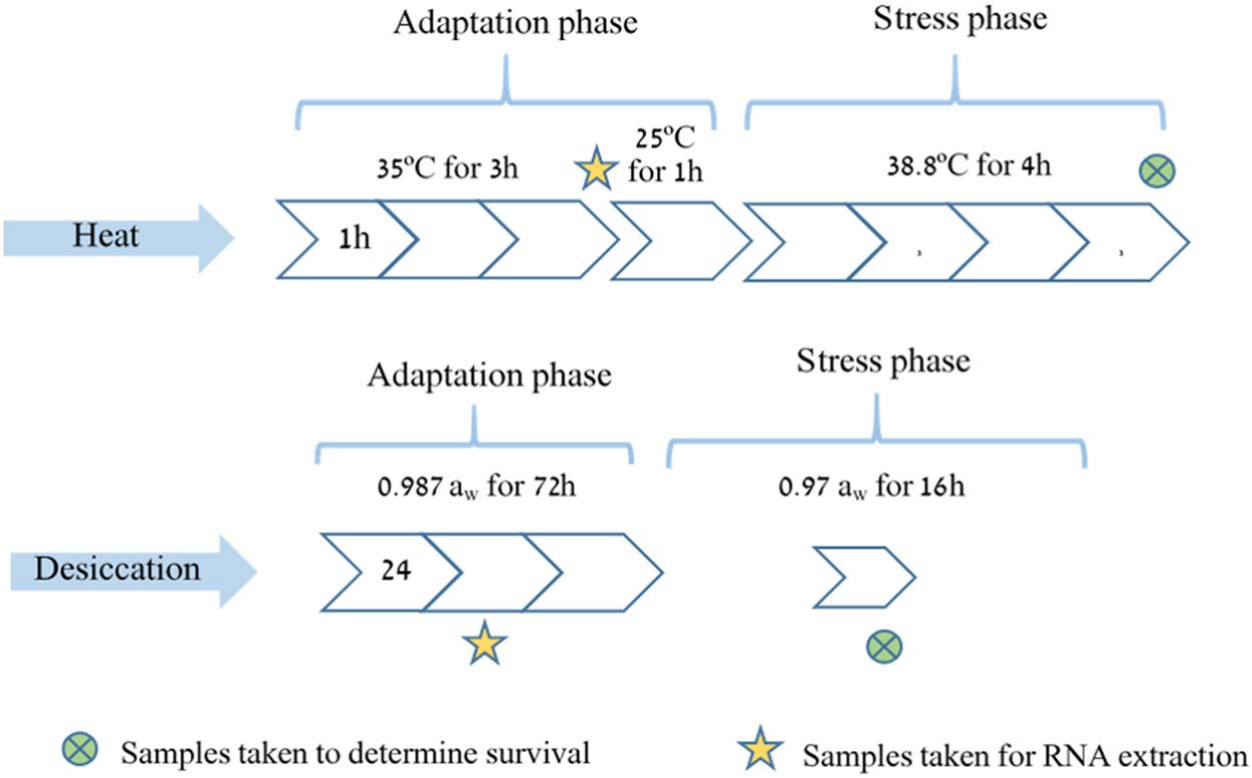
Experimental procedure of the stress assays, including timing of survival and gene expression samplings. a_w_ = active water.

Gradient temperature PCR program was designed in order to determine the survival rates of the commercial nematode line, EN-01, under increasing temperatures. The program was planned as follows: adaptation phase of 3 hours at 35 °C, recovery phase of 1 hour at 25 °C, stress phase of 4 hours at gradient temperatures as follows: 37 °C, 37.4 °C, 37.7 °C, 38.2 °C, 38.7 °C, 39.4 °C. After the stress phase, an additional recovery phase was performed at 25 °C overnight^47^. Control tubes kept at 25 °C during the heat stress phase. The experiment was performed in PCR tubes with ~200 IJs per tube. All treatments were performed with a single batch of infective juveniles and repeated in three biological replicates. The experiment was repeated three times. Nematodes survival rate was determined for each treatment at the end of the experiment by counting the number of live and dead nematodes under a stereomicroscope (See scheme Fig. 8).

### Desiccation tolerance bioassay – Gradient active water

Polyethylene glycol 600 (PEG 600, Sigma-Aldrich) dilutions were used to create gradient desiccation conditions by the hygroscopic method. PEG is a clear, non-toxic and non-ionic, hygroscopic solution^46^. Desiccation stress was measured as water activity (a_w_ value) in each PEG dilution. Water activity is defined as the relative proportion of unbound water molecules in a sample. The lower the water activity of a solution is, the higher the desiccation is. IJs of EN-01 were subjected to the different PEG dilutions in order to determine their survival under increasing desiccation conditions. Initially, 3000 IJs were exposed to adaptation phase in 15 ml tubes containing 10% v/v PEG (0.987 a_w_). The tubes shaken at 155 rpm for 72 hours at 25 °C. After adaptation, tubes were centrifuge in 5000 rpm for 5 minutes in order to precipitate the nematodes. The supernatant was discarded and IJs were re-suspend in 2 ml ringer buffer (9 gl-1 NaCl, 4.42 gl-1 KCl, 0.37 gl-1 CaCl2 ×2 H2O, 0.2 gl-1 NaHCO3) in order to wash the PEG solution. Wash step was performed three times. In the third time the supernatant was discarded, control IJs were re-suspended in 2 ml water, and the treated ones were suspended in 2 ml of 20% v/v (0.98 a_w_), 30% v/v (0.975 a_w_), 33% v/v (0.971 a_w_), 35% v/v (0.968 a_w_), 37% v/v (0.965 a_w_), 40% v/v (0.96 a_w_) and 50% v/v (0.93 a_w_) PEG solution for 16 hours at 25 °C, shaken at 155 rpm. Nematode survival percentage was determined for each treatment by counting the number of live and dead nematodes under a stereomicroscope at the end of the experiment (See scheme Fig. 8). All treatments were performed with a single batch of infective juveniles and repeated in three biological replicates. The experiment was repeated three times.

### Comparative heat tolerance bioassay - constant temperature

Two hundreds IJs from each nematode isolate and the commercial line, EN-01, were subjected to heat tolerance bioassay in PCR tubes. PCR program was set as follows: adaptation phase of 3 hours at 35 °C, recovery phase of 1 hours at 25 °C, stress phase of 4 hours at 38.8 °C (the determined MT °C_50_ of EN-01), recovery phase at 25 °C overnight. Control tubes were kept at 25 °C during the heat stress phase. Each isolate had three biological replicates and the experiment was repeated three times. Nematodes survival percentage was determined for each isolate at the end of the experiment by counting the number of live and dead nematodes under a stereomicroscope.

### Comparative desiccation tolerance bioassay – constant active water

Three thousands IJs from each nematode isolate and the commercial line, EN-01, were subjected to 15 ml tubes in a 10% v/v PEG solution for 72 hours in order to apply adaptation for desiccation stress. After adaptation, centrifugation and wash were carried out similarly to the gradient desiccation experiment. In the third wash, the supernatant was discarded, the control IJs were re-suspended in 2 ml water, and the treatment ones were re-suspend in 2 ml of 33.8% v/v PEG (the determined MW_50_ of EN-01). Tubes were kept for 16 hours at 25 °C, shaken at 155 rpm. Each isolate had three biological replicates and the experiment was repeated three times. Nematodes survival percentage was determined for each isolate at the end of the experiment.

### Preparation of total RNA

Gene expression patterns were studied in isolates with contrasting phenotype of heat stress tolerance. The commercial line, EN-01 (e-nema, GMBH, Schwentinental, Germany) was used as a reference strain for studying the gene expression patterns under stress conditions. Samples were taken at early stages of exposure to stress (Fig. 8) as the main events of gene expression take place at early stages of detection of stress by the nematodes^29^. Heat stress samples were taken after exposure to 35 °C for 3 hours. Three biological replicates performed for each condition. The control consisted of non-heated nematodes from the same batch heated counterparts, kept in distilled water at 25 °C. After the exposure period, samples were immediately frozen in liquid nitrogen and dried in a lyophilizer. Freeze-dried samples were ground to a fine powder and total RNA extracted from the desiccated, heated and control samples using the Plant/Fungi Total RNA purification Kit (Norgen Biotek Corp.) according to the manufacturer’s instructions. Analysis was done in the Core Genomics Facility University of Illinois at Chicago. From the total RNA, the mRNA was isolated using selective Poly (A) column. cDNA libraries were enriched by PCR using NuGen Universal Plus mRNA chemistry kit according to the manufacturer’s instruction. Cluster generation and sequencing of the cDNA transcripts were performed on a single lane of NovaSeq SP flow cell.

### Transcriptome analysis

Homogenous inbreed line of EN-01, IL-3, was used as a reference genome in the present study (Unpublished genome). Reads were aligned to IL-3 genome using Tophat2 software (v2.1). Gene abundance estimation was performed using Cufflinks (v2.2) combined with gene annotations from previous study^56^.

Differential expression analysis was completed using the DESeq. 2 R package. Genes that varied from the control more than twofold, with an adjusted *P*-value of no more than 0.05, were considered differentially expressed. Venn diagrams were calculated using “Venny” tool^57^. The transcriptome was used for a search of the NCBI non-redundant (nr) protein database, employing the DIAMOND program^58^. The results were exported to Blast2GO version 4.0^59^ for gene ontology (GO) assignments. The eggNOG-mapper v2 tool) http://eggnog-mapper.embl.de/ (was used for functional annotation. The KAAS tool (Kegg Automatic Annotation Server; http://www.genome.jp/tools/kaas/) was used for KEGG ontology and KEGG pathway assignments. Gene ontology enrichment analyses was carried out using Blast2GO^59^ software based on Fisher’s Exact Test^60^. The ReviGO web server was used for visualization of the GO terms in a semantic similarity-based scatterplot [http://revigo.irb.hr]^61^.

### Genome sequencing

Isolates of the specie *H. bacteriophora* with varying degrees of tolerance to each of the stress conditions were chosen for genome sequencing in order to search for polymorphism in the genome in relation to the tolerance capabilities. Two-hundred μl (~50 ng/μl) of genomic DNA of each isolate were prepared and sent for sequencing. Macrogen Inc. (South Korea) performed the genome sequencing using TruSeq DNA PCR free for library construction and NovaSeq. 6000 for the sequencing.

### SNPs calling on genomic sequences

Homogenous inbreed line of EN-01, IL-3, was used as a reference genome in the present study (Unpublished genome). Reads were aligned to IL-3 genome using Tophat2 software (v2.1) using default parameters. Gene abundance estimation was performed using Cufflinks suite (v2.2) by the Cuffquant combined with gene annotations from previous study 57, and then Cuffnorm was used for the normalization. Paired-end reads of the 3 samples were mapped to the reference genome (IL-3) using the BWA mem program with default parameters^62^. The resulting mapping file was processed using Picard tool (http://broadinstitute.github.io/picard/; version 1.95) for adding read group information, sorting, marking duplicates, and indexing. Then, the local re-alignment process for locally re-aligning reads such that the number of mismatching bases is minimized across all the reads was performed using the Realigner Target Creator of the Genome Analysis Toolkit version 3.4–0 [GATK; version http://www.broadinstitute.org/gatk/]^63^. Finally, the variant calling procedure was performed using Haplotype Caller of the GATK toolkit, for the detection of SNPs between the variants and the reference IL-3 genome. Only homozygous SNPs were further analyzed. An in-house Perl script was used to define SNPs in genes or upstream/downstream to genes (1000 bp) and the define stop codon or non-synonymous substitutions.

### Statistical analysis

All the statistical analysis was done using JMP, Version 14. SAS Institute Inc., Cary, NC, 1989–2019. Results are presented as mean ± SE of replicate analysis and are either representative of or include at least three independent experiments. Means of replicates were subjected to statistical analysis and considered significant when P < 0.05.

To determine Mean Tolerated Temperature (MT°C_50_) and Mean Water Activity (MW_50_) The mean percentages of survival in each treatment of heat or desiccation submitted to a Probit test with inverse prediction probability of 0.5 in order to find the predicted temperature and predicted active water value in which the survival of the commercial line, EN-01, is 50%, referring to MT °C_50_ and MW_50_ respectively. For comparative analysis of the heat and desiccation tolerance, the square roots of the proportion data were arcsine transformed and then subjected to ANOVA test to determine the percentage survival by strain of nematode. A multiple comparison, Dunnett with EN-01 as control was done in order to compare the survival rates of the different isolates with the commercial line.

## Data Availability

All sequencing results were deposited in NCBI SRA under BioProject number PRJNA607179 BioSample accession numbers SAMN14123596, SAMN14123595, SAMN14123594, SAMN14123593, SAMN14123635, SAMN14123634, SAMN14123633.
